# Impact of Aggregation Pheromone Traps on Spatial Distribution of *Halyomorpha halys* Damage in Apple Orchards

**DOI:** 10.3390/insects15100791

**Published:** 2024-10-11

**Authors:** Veronica Carnio, Riccardo Favaro, Michele Preti, Sergio Angeli

**Affiliations:** 1 Faculty of Agricultural, Environmental and Food Sciences, Free University of Bozen-Bolzano, 39100 Bolzano, Italy; veronica.carnio@unibz.it (V.C.); riccardo.favaro@unipd.it (R.F.); 2 Department of Chemical Sciences, University of Padova, 35131 Padova, Italy; 3 Astra Innovazione e Sviluppo, 48018 Faenza, Italy; michele.preti@astrainnovazione.it; 4 Competence Centre for Plant Health, Free University of Bozen-Bolzano, 39100 Bolzano, Italy

**Keywords:** brown marmorated stink bug, attract and kill, mass trapping, invasive species, dispersive distance, integrated pest management

## Abstract

**Simple Summary:**

*Halyomorpha halys* is a major pest of concern for many tree crops, such as pome and stone fruits. Currently, the management of this pest relies mainly on insecticides. The male-produced aggregation pheromone of *H. halys* showed promising results in attracting adults and nymphs, opening possibilities to develop new low-input insecticide management techniques. Our study demonstrated that traps baited with *H. halys* aggregation pheromone, when placed along the borders of an apple orchard, caused a shift in the spatial distribution of fruit damage. Although the overall fruit damage was not reduced, fruit damage significantly concentrated near the border treated with the traps. Limiting insecticide treatments to a small area near the orchard border, where insects are aggregated, can maximize the efficacy while reducing relevant side effects.

**Abstract:**

*Halyomorpha halys* (Stål) (Hemiptera: Pentatomidae) is an invasive pest causing significant damage to tree crops. Our study examined the impact of newly designed aggregation pheromone-baited ‘mini–sailboat’ (MSB) traps for controlling *H. halys* and its effect on the spatial distribution of fruit damage. Four replicates of four traps, with a total of 16 MSB traps, were placed along a 1.3 km border of apple orchards, concentrating the traps on one side of the orchards. A fruit damage assessment for incidence and severity was conducted at increasing distances from the orchard border where the traps were placed, encompassing 107 assessment points. Our study showed that deploying MSB traps along the orchard border significantly increased fruit damage within the first 45 m compared to control plots without traps. However, beyond the first 45 m from the border, there was a significant reduction in damage incidence. In the treated plots, 50% of the damage occurred within 26 m of the traps, while in control plots, within 85 m. Shifting the fruit damage pattern means restricting the pests lingering in a narrow strip near the MSB traps, which paves the way for improved techniques to restructure the crop perimeter.

## 1. Introduction

**Figure 1:**
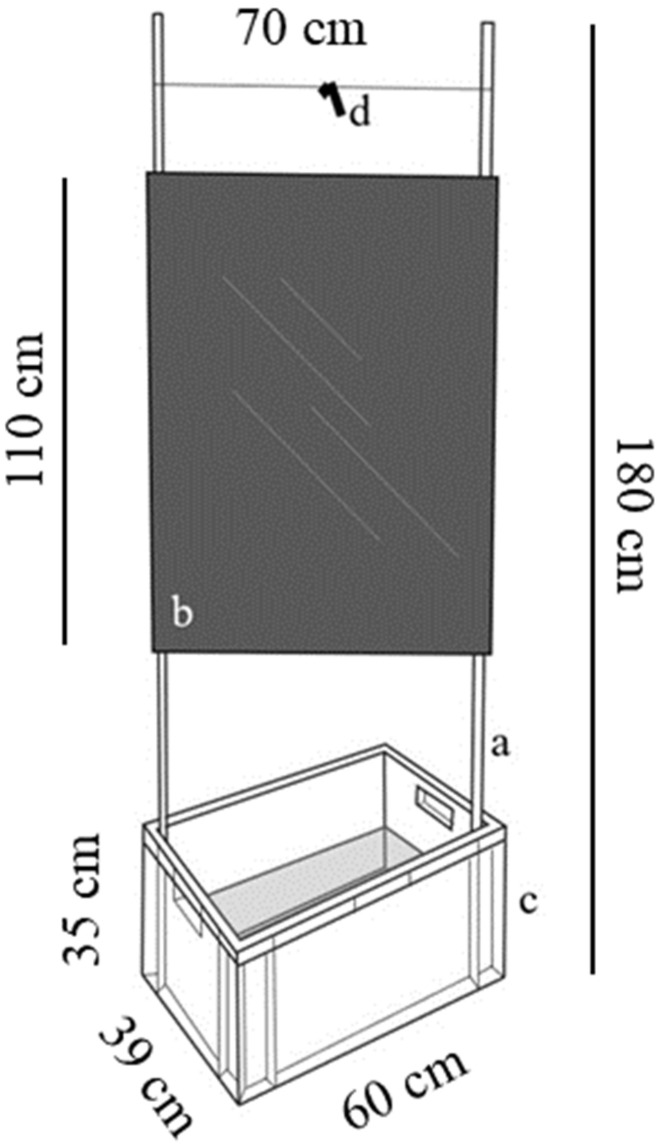
Schematic representation of mini–sailboat trap prototype consisting of (**a**) rectangular bamboo structure, (**b**) black sticky nylon sail, and a (**c**) plastic bin, filled with soapy water, placed at the base of the structure. At the top of the trap, (**d**) the commercial monitoring aggregation pheromone lure for *H. halys* (PHEROCON BMSB DUAL, Trécé Inc., Adair, OK, USA) is hung.

*Halyomorpha halys* (Stål, 1855) (Hemiptera: Pentatomidae) is an invasive pest that originated in eastern Asian countries and has now spread to the USA, South America, and Europe. Specifically, in northern Italy severe infestations and damage have been recorded extensively [1]. The damage results from the feeding activity on fruit or seeds by both adults and nymphs of *H. halys*. The feeding occurs on a wide range of host plants, especially fruit tree crops such as pome fruit, stone fruit, and grapes [1]. Additionally, it affects corn, soybean, and vegetables, as well as ornamental and wild tree and shrub species such as maple, linden, elm, and hazel [2]. High mobility and a wide-ranging diet enable *H. halys* to spread to new areas and to avoid control measures quickly [3]. *Halyomorpha halys* management relies mainly on broad-spectrum insecticides [4]. The invasion of this species led to the overuse of synthetic insecticides, disrupting low-impact IPM practices traditionally applied for other pests and causing secondary pest outbreaks [5]. Current IPM strategies explored for the management of *H. halys* include perimeter restructuring techniques in apple orchards [3], application of sulfur-based products [6], exclusion nets [7], behavioural manipulation [8], classical biological control [9], and various attract and kill (AK) approaches [10,11,12,13,14,15]. The commercial lure commonly used to monitor *H. halys*, namely PHEROCON BMSB DUAL Lure (Trécé Inc., Adair, OK, USA), is loaded with the male-produced aggregation pheromone and is based on a combination of the murgantiol stereoisomers (3*S*,6*S*,7*R*,10*S*)-10,11-epoxy-1-bisabolen-3-ol and (3*R*,6*S*,7*R*,10*S*)-10,11-epoxy-1 bisabolen-3-ol [16], along with methyl (2*E*,4*E*,6*Z*)-decatrienoate (MDT), which acts as a pheromone synergist [17]. It effectively attracts both adults and nymphs of *H. halys* throughout the season in a dose-dependent manner and has been extensively validated across different geographical regions [18]. This aggregation pheromone-based lure is an optimal candidate for implementing AK techniques, where insects are attracted in a defined space and subsequently suppressed with insecticides. Most AK studies involved applying attractants and insecticides directly to host plants or using long-lasting insecticidal nets (LLINs) loaded with active ingredients such as alpha-cypermethrin or deltamethrin. These studies have shown variable effectiveness and were impacted by regulatory changes, such as the withdrawal of alpha-cypermethrin in the EU [10,11,12,15]. Mass trapping (MT), with the killing method consisting of a physical retention medium rather than an insecticide, offers a more sustainable and adaptable solution under these constraints. Various trap designs are used for *H. halys*, such as pipe traps, hanging pyramid traps, yellow sticky traps, orange delta traps, ground-deployed standard black pyramid traps [18,19]. However, these traps are designed predominantly for *H. halys* monitoring and not MT. For this reason, this study focused on ‘sailboat traps’ [13], which utilize a cost-effective combination of glue panels and water traps, because of their wide retention surface. Interest in sailboat traps has grown in recent years, leading several Italian growers to independently construct similar structures for their farms in a first attempt of *H. halys* MT [13,14]. The aggregation pheromone-based trap proposed in this study, named a “mini–sailboat” (MSB) trap (Figure 1), is composed of a large black sticky panel acting as a “boat sail” and a water bin acting as the “boat hull”. This design provides two advantages for mass trapping over the standard monitoring traps: a more attractive visual cue [20] and a larger trapping surface.

This study aimed to collect empirical data about the feasibility of *H. halys* MT with MSB traps and the effect on fruit damage in continuous expanses of apple orchards.

## 2. Materials and Methods

### 2.1. Trap Design

The newly designed MSB trap, proposed in this study, consisted of a bamboo frame supporting a black nylon sail measuring 110 cm in height and 70 cm in width (Figure 1). A silicone glue (‘Insect catcher spray’ Siliconi Chimica srl, Marostica, Italy) was applied in spray form to both sides of the nylon sail to obtain a uniform sticky surface. The base of the structure was a plastic bin (32 cm in height, 60 cm in width, and 39 cm in depth) filled with soapy water to ca. 20 cm. The sail had a retention surface area (considering both sides) of 15,400 cm^2^ and the bin contributed an additional 1920 cm^2^, giving a total retention surface area of ca. 17,320 cm^2^ per trap. The commercially available aggregation pheromone lure for *H. halys* monitoring (PHEROCON BMSB DUAL, Trécé Inc., Adair, OK, USA) was placed above the sail to facilitate the spread of the pheromone plume around the MSB trap. This lure consists of two black PVC dispensers loaded with murgantiol and MDT, respectively.

### 2.2. Field Sites

The field study was carried out during the 2023 season at two commercial apple orchards in South Tyrol, Italy. One was located in Postal (BZ), with a size of approximately 40 ha (bounding box 46.6194° N, 11.1796° E; 46.6117° N, 11.1866° E). The other was located in Lana (BZ), with a size of approx. 4.3 ha (bounding box 46.5831° N, 11.1684° E; 46.5798° N, 11.1728° E). These apple orchards are located in the central part of the Adige valley within South Tyrol, one of the most significant apple-growing regions in Europe. This area consists of an almost continuous expanse of apple orchards, covering approx. 17,000 ha, extending approx. 100 km in length, and an average of 2 km in width. It is bordered by a strip of vineyards covering the surrounding mountain slopes. The orchard locations were selected considering the prevalent wind direction (North to South in 2023 [21]) and the presence of natural landscape features that served as physical barriers on the longest border of the orchards. This was performed to avoid insect migration inside the orchards under study from the opposite side. The apple orchards were managed according to either integrated [22] or organic farming [23] regulations, as provided by the local growers’ consultancy organization, Südtiroler Beratungsring, and featured a diverse array of apple varieties (e.g., Kanzi^®^, Gala, Rosy Glow, Granny Smith, Red Delicious, etc.) with a harvest period ranging between September and October.

### 2.3. Border Trapping Trials

**Figure 2:**
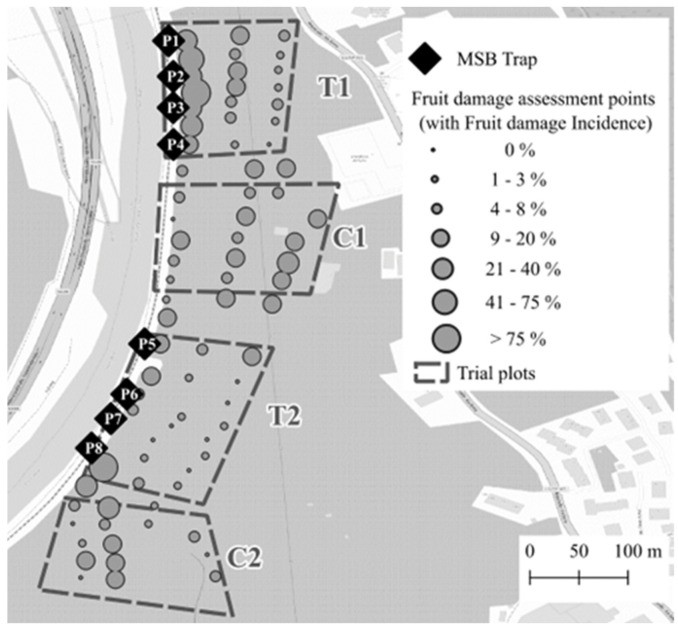
Experimental design at Postal site (South Tyrol, Italy). Treatment plots with 4 mini–sailboat traps each (T1, T2) and control plots (C1, C2) are indicated by a dashed line. The 8 mini–sailboat (MSB) traps locations along the apple orchards border are marked with black diamonds and identification codes. Fruit damage assessment points are represented by dots of different sizes, indicating fruit damage incidence from 0% (small) to 100% (big).

At the Postal site, four plots, each measuring two hectares (200 m of the orchard border’s front side × 100 m from the border to the inner part of the orchard), were selected. Two plots were equipped with a set of four MSB traps each (Figure 2, T1 and T2) to MT *H. halys*, while the other two plots served as control plots without traps (Figure 2, C1 and C2). The treated and control plots were arranged alternately, with the control plots located at least 80 m from the nearest MSB trap to avoid any interaction with the treatment plots. Within the MT-treated plots, the MSB traps were spaced 40–50 m apart following Leskey et al. [18], along the western border of the apple orchard, adjacent to a riverbank on the opposite part of the orchard’s border (approx. 7 m apart from the orchard).

**Figure 3:**
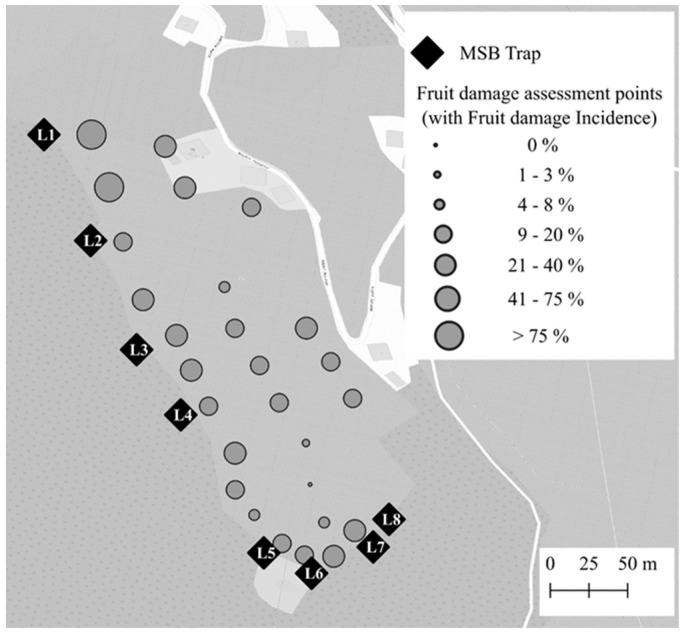
Experimental design at Lana site (South Tyrol, Italy). The whole apple orchard cluster was considered as a unique treated plot. The 8 mini–sailboat (MSB) trap locations are marked with black diamonds and identification codes. Fruit damage assessment points are represented by dots of different sizes, indicating fruit damage incidence from 0% (small) to 100% (big).

At the Lana site, only one large plot treated with MSB was established, as the reduced size of this area (approx. 4.3 ha) and commercial operations did not allow for an additional control plot. In this plot, eight MSB traps were spaced 30–90 m apart along the western border of the apple orchard due to the landscape characteristics of this site, with a crag in one side of the orchard (Figure 3). At this site also, the MSB traps were set outside the orchards at a distance of ca. 7 m from the nearest apple tree.

All traps were deployed on 2 May 2023 at both sites. Trap maintenance involved replacing the silicone glue every 2–3 weeks to ensure the adhesive capacity of the nylon sail and refilling of the soapy water in the plastic bins when needed. Pheromone lures were replaced according to the 12-week product longevity provided by the manufacturer.

### 2.4. Insect Catch Assessment

This study was carried out in spring–summer 2023, and the MSB traps were monitored six times from 17 May to 2 September 2023 (17 May, 8 June, 14 July, 26 July, 11 August, and 2 September). For each trap, the number of individual nymphs, adult males, and females of *H. halys* was recorded, considering separately the individuals stuck on the sail and drowned in the soapy water within the bin. All insects were removed from the trap at each counting period. Non-target catches were also collected but only other pentatomids were identified and counted to assess the trap selectivity. Data for these insects were collected in the same manner as for *H. halys.*

### 2.5. Fruit Damage Assessment

The assessment of fruit damage caused by *H. halys* was carried out during the pre-harvest period on 12–16 August 2023. At the two sites, a total of 107 assessment points within the apple orchards were chosen at increasing distances from the border towards the opposite border of the apple orchards (Figure 2 and Figure 3) in both the plots with traps and without traps. Each assessment point was comprised of 4 apple trees on one side of the inter-row and 4 apple trees on the opposite side. The tree damage assessment was conducted up to a canopy height of 180 cm, starting from the plant bottom and moving upwards. For each assessment point, approx. 90 (92 ± 0.2) fruit were observed. The feeding injuries were diagnosed as being caused by *H. halys*, following Morrison et al. [10]. Fruit were classified for their damage severity into six categories based on the number of feeding injuries caused by *H. halys* per each fruit. For each class, a corresponding damage coefficient (*V*) was assigned. The classification system was as follows: Class 1 indicates no damage (*V* = 0); Class 2 indicates one injury (*V* = 1); Class 3 indicates 2–3 injuries (*V* = 2.5); Class 4 indicates 4–6 injuries (*V* = 5); Class 5 indicates 7–10 injuries (*V* = 8.5); and Class 6 indicates 11–20 injuries (*V* = 15.5). The damage coefficients made it easier to provide a numerical value to quantify the overall fruit damage severity (see Supplementary Materials, Table S1). Fruit assessment was non-destructive, and the visual observations considered external visible symptoms to count the number of feeding damages per fruit. To validate the visual damage assessment, 10% of the fruit were randomly selected, collected, and peeled to verify the internal symptoms.

The fruit damage incidence (1) and the fruit damage severity (2) were calculated for each assessment point:

(1) $$\mathrm{Fruit}\;\mathrm{Damage}\;\mathrm{Incidence}\left[\%\;\mathrm{of}\;\mathrm{damaged}\;\mathrm{fruit}\right]=\;\frac{\mathrm{number}\;\mathrm{of}\;\mathrm{injured}\;\mathrm{fruit}}{\mathrm{total}\;\mathrm{number}\;\mathrm{of}\;\mathrm{assessed}\;\mathrm{fruit}}*100$$

(2) $$\mathrm{Fruit}\;\mathrm{Damage}\;\mathrm{Severity}\left[\mathrm{average}\;\mathrm{number}\;\mathrm{of}\;\mathrm{injuries}\;\mathrm{per}\;\mathrm{injured}\;\mathrm{fruit}\right]=\frac{\sum_{1}^{i}N_{i}V_{i}}{\sum_{2}^{i}N_{i}}$$

where N_i_ is the number of affected fruit belonging to the i-th class for the damage assessment point and V is the damage coefficient.

### 2.6. Spatial Analysis

The locations of MSB traps and the damage assessment points were recorded in the field using Xiaomi 12T Pro (Xiaomi, Beijing, China) by means of the ‘Mergin maps’ application [24]. The distance matrices between the MSB trap positions and the damage assessment points were calculated by the QGIS analysis tool “distance matrix” [25]. To calculate the distances between the orchard border where the traps were placed and the damage assessment points, we first created two lines that interpolated all the 28 recorded points along the orchard border at the Postal site and the 15 points along the orchard border at the Lana site. Next, we calculated the minimum distances between the line and all the damage assessment points for each site.

### 2.7. Catch Index

For each assessment point, the weighted contribution of the MSB traps was obtained by dividing the total number of *H. halys* catches in the traps by their relative distance to each of the assessing points to obtain a catch index (3) according to the equation:

(3) $$\mathrm{Catch}\;\mathrm{index}=\sqrt{\sum_{1}^{i}{\left(\frac{H_{x}}{d_{xi}}\right)}^{2}}$$

where *i* was the assessment point, $H_{x}$ the number *H. halys* individuals per ${trap}_{x}$, and $d_{xi}$ the distance from the trap and the assessment point *i*, hence providing a weighted estimation of the number of caught insects on each assessment point. The square root sum of the squared ratio between trap catches and distance emphasizes the impact of both high insect catches and shorter distances while penalizing combinations of low catches and longer distances. Hence, the closer to a trap with many catches, the higher the catch index (see Supplementary Materials, Table S2).

### 2.8. Statistical Analysis

The data were analysed using the package R version 4.3.1 (R Core Team, 2023).

(1) The catch data collected during the season, divided by insect sex and distributed between the trap sail and the trap bin, were analysed with a generalized mixed-effect negative binomial model (package “MASS”) [26], considering the site, the sex, and the trap part (sail or bin) as explanatory variables and the nested date and the trap ID as random variables. A negative binomial model was used since the data are count-based and over dispersed, meaning that the variance exceeds the mean, which a Poisson model cannot handle effectively [27]. A pairwise comparison between female and male catch was performed between sail and bin (packages “emmeans”, [28]).
(2) The *H. halys* catch within the two sets of traps in the Postal site and between the locations of the Postal and Lana sites were tested for differences by one-way ANOVAs. The number of catches was also tested to evaluate a possible effect of the trap vicinity to the control plots: traps were assigned a progressive number (1 the closest, 4 the further) and the catches were tested in a linear model.
(3) At the Postal site, the distribution of the damage incidence was analysed with a negative binomial model, using the catch index, the distance to the nearest trap, the apple variety, the presence of traps and the management (organic or IPM) and their interaction, and the presence of traps and the distance to the border and their interaction as explanatory variables. Terms that did not significantly contribute to the model were discarded, and the model with the lowest AIC was retained. The damage severity was analysed following the same approach using the catch index, the distance to the nearest trap, the apple variety, the presence of traps and the management (organic or IPM) and their interaction, and the presence of traps and the distance to the border and their interaction as explanatory variables. The area under the fitted curves was integrated to calculate the cumulated damage incidence, which was used to extrapolate the distance point at which the cumulated damage incidence reached 50%. The variation in damage incidence in the presence of traps was calculated by subtracting the damage incidences estimated by the model curves. For each distance value, the estimated incidence in presence traps was subtracted from that of control plots, resulting in a differential effect of the traps.
(4) At the Lana site, the damage incidence was analysed with a generalized linear model specifying a Poisson distribution, since the data were count-based and the mean and variance were approximately equal. The number of injured fruit was considered as a response variable, while the number of fruit observed was log-transformed as an offset. The distance from the border, the apple variety, and the distance from the nearest trap were considered as explanatory variables. The damage severity was analysed with a negative binomial model, and the distance from the border, the apple variety, and the distance from the nearest trap were considered as explanatory variables.

The residuals of the fitted model were checked for normality, overdispersion, deviation of quartiles, and outliers aided by the package “DHARMa” [29]. For each model, a Pearson’s chi-square goodness of fit test on the residuals fitting was performed to provide a single, clear value of the model reliability. The significance of the model terms was estimated with an analysis of deviance (type III in Postal, type II in Lana) using the package “car” [30]. The package “ggplot2” [31] was used for the graphics. Data in the text are reported as mean values ± standard deviation.

## 3. Results

### 3.1. Trap Catches Evaluation

**Figure 4:**
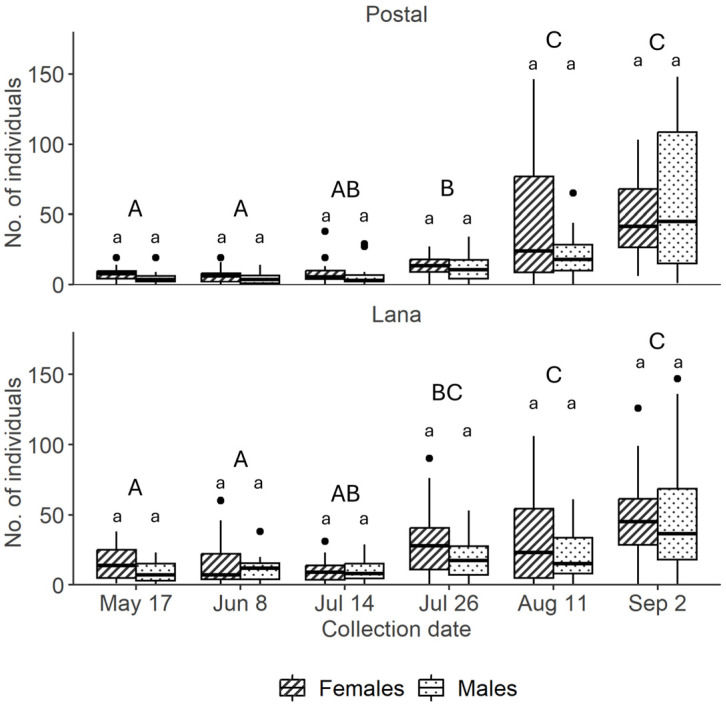
Box and whisker plot of the total number per site of *Halyomorpha halys* adults collected from the mini–sailboat traps during the 2023 season (17 May, 2 September, 2023). Boxplot fill pattern indicate the sex (males or females) in the two apple orchards at the Postal and Lana sites (South Tyrol, Italy). Lowercase letters indicate difference between the sex of caught *H. halys*, and capital letters indicate difference between dates.

**Figure 5:**
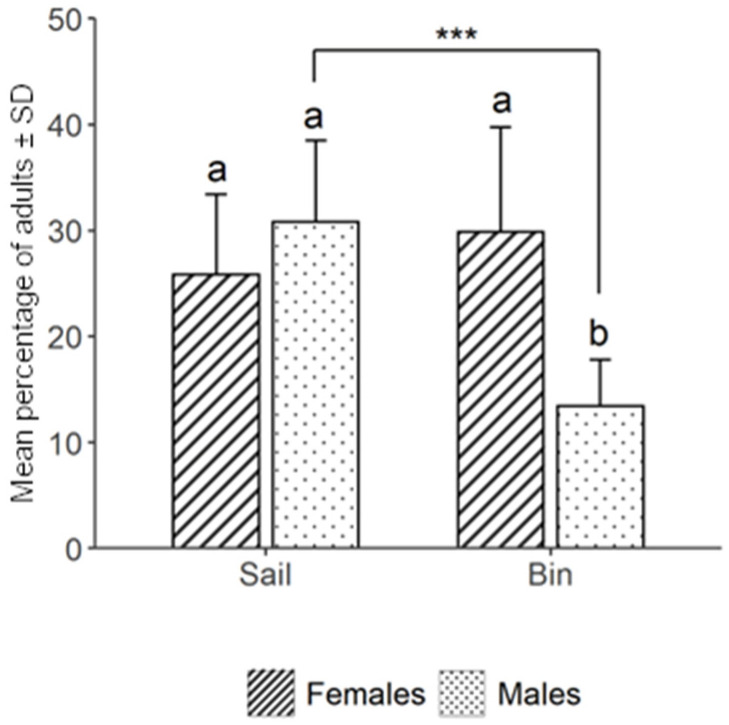
Mean percentage (±SD) of cumulated *Halyomorpha halys* adult females and males per mini–sailboat trap throughout the whole trial duration in both sites, Postal site and Lana (South Tyrol, Italy). Data are shown separately for the part of the trap in which insects were recovered (sail and bin) from 17 May to 2 September 2023. Letters show statistical differences after pairwise comparison between males and females in sail or bin. The asterisks indicate statistical significance (*p* < 0.001).

In our study, the average number of *H. halys* adults caught per trap over the period was 520.12 ± 241.38 (see Supplementary Materials, Table S2). The total number of *H. halys* nymphs caught across the whole study was considered to be insufficient for further analyses, with only 44 individuals recorded in the MSB traps. The average number of adult catches per trap at the Postal site was 481.5 ± 125.3, which did not differ from the catches at the Lana site, which averaged 558.7 ± 325.1 (F_1,14_ = 0.39, *p* = 0.540) (Figure 4). The statistical analyses of the sex ratio of catches, and the trend of catches during the season, confirm that there was no difference between the two sites (χ^2^_1,378_ = 2.77, *p* = 0.110). The trap catches of *H. halys* adults increased during the season (χ^2^_5,378_ = 174.48, *p* < 0.001), with a more conspicuous number of adults recovered in the late summer (Figure 4). Overall, sex has a significant impact on the catch rates (χ^2^_1,378_ = 43.15, *p* < 0.001), with an average of 238.97 males against 281.15 females caught per trap (in total 3695 males and 4627 females) (Figure 5). This resulted in a male-to-female sex ratio of 0.85:1. However, the *H. halys* male and female catches did not differ within each of the six dates on which catch data were recorded, likely due to the high data variability. In the MSB, the two trap parts (sail and bin) caught a comparable number of adult insects (Figure 5), considering each of the 16 traps independently with 56.68% ± 13.93 of the insects caught on the sticky sail and 43.31% ± 13.93 in the bin with water (χ^2^_1,378_ = 2.59, *p* = 0.107). Females were caught in the same number by both the sail and the bin, with 47.07% ± 16.72 of insects caught by the sail and 52.92% ± 16.72 caught by the bin (χ^2^_1,378_ = 5.27, *p* = 0.380). However, males were significantly more on the sail than in the bin, with 68.90% ± 10.67 males caught by sail and 31.9% ± 10.67 males caught by the bin (χ^2^_1,378_ = 32.16, *p* < 0.001) (Figure 5). The pairwise comparison revealed that in the bin, *H. halys* males were significantly less frequent than females (z = 6.56, *p* < 0.001).

At the Postal site, the average number of adult catches per trap did not differ significantly between the two plots, with 425.5 ± 141 for plot T1 and 537.5 ± 91.7 for plot T2 (F_1,6_ = 1.77, *p* = 0.230). Notably, traps located near the control plots (Figure 2, P4, P5, P8) did not catch more insects than the other traps (F_1,6_ = 0.39, *p* = 0.580).

In total, across both the Postal and Lana sites, 34 non-target pentatomids were recorded among the bycatches, including both adults and nymphs caught during the trial. These comprised 23 *Nezara viridula* L., 7 *Eysarcoris* spp., 2 *Graphosoma italicum* Müller, and 2 *Pentatoma rufipes* L. These non-target pentatomids represented only 0.004% of the total pentatomids (including *H. halys*) caught by the traps (8356) and were therefore not considered for further analyses.

### 3.2. Analysis of Fruit Damage Caused by H. halys at Postal Site

**Figure 6:**
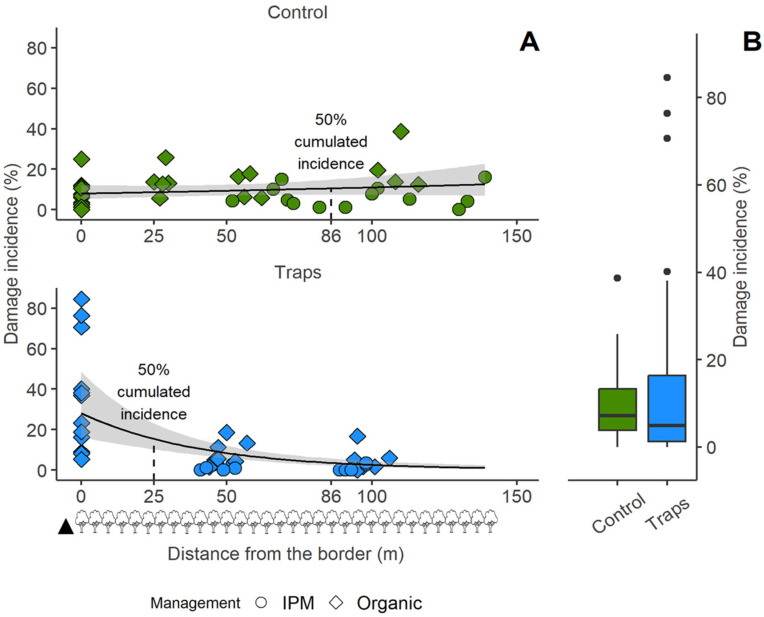
Apple fruit damage incidence caused by *Halyomorpha halys* in the control plots and in the plots with mini–sailboat (MSB) traps at the Postal site (South Tyrol, Italy). (**A**) Values plotted against the distance from the orchards border. Dot shape shows whether the parcels were managed organically or according to IPM practice. The lines show the trends, and the grey ribbons display 0.95 confidence intervals around the smooth. The black triangle at the bottom represents the MSB trap location, while the tree symbols indicate the plant rows in the apple orchard (space between the rows is ~4 m). Half of the total fruit damage in the plots lies under the curves between 0 and the reported distance of 50% cumulated incidence. (**B**) Overall fruit damage incidence in control plots and in the plots with MSB traps in the Postal trial.

The fruit *damage incidence* was correctly estimated by the model (see Supplementary Materials, Table S3) (model Pearson’s χ^2^ = 79.08, *p* = 0.29). The catch index was not significant, and it was discarded by the model, meaning that the amount of *H. halys* caught by each trap was not a relevant predictor of the damage incidence. Also, the apple variety was a not significant predictor. On the other hand, the distance from the border resulted in being very important, and in the plots with traps it considerably decreased as the distance increased (χ^2^_1,71_ = 28.04, *p* < 0.001) (Figure 6). The distance to the nearest trap was not significant for the control plots (χ^2^_1,71_ = 1.55, *p* = 0.210), showing that the assessment points in the control plots were not affected by the vicinity of a trap, but that they were instead relevant for the plots with traps (χ^2^_1,71_ = 5.40, *p* = 0.020), with the fruit damage incidence increasing when closer to the traps.

The total fruit damage incidence was not affected by the presence of the traps (χ^2^_1,71_ = 0.35, *p* = 0.551), and on average it was overall 9.44% ± 8.15 in the control plots and 13.69% ± 21.47 in the plots with the MSB traps. The higher average and variability of the damage incidence in the presence of traps is clearly visible in Figure 6B, which displays an abrupt shift in the fruit damage incidence towards the MSB traps (Figure 6A), with no appreciable incidence reduction overall. The parcels composing the experimental orchards were managed with both organic and IPM approach, and this was considered in the analysis. The management significantly affected the fruit damage incidence (χ^2^_1,71_ = 7.47, *p* = 0.006), as it was greater in the assessment points belonging to organic parcels, with a mean fruit damage incidence of 15.18% ± 18.29, than in those of IPM-managed ones, with a mean fruit damage incidence of 3.77% ± 4.66. The distances at which the cumulated fruit damage incidence reaches 50% (i.e., half of the estimated total incidence lies between the orchard border and that given distance) varied greatly between control plots and plots with traps, respectively, 86.2 m and 25.6 m (Figure 6A).

**Figure 7:**
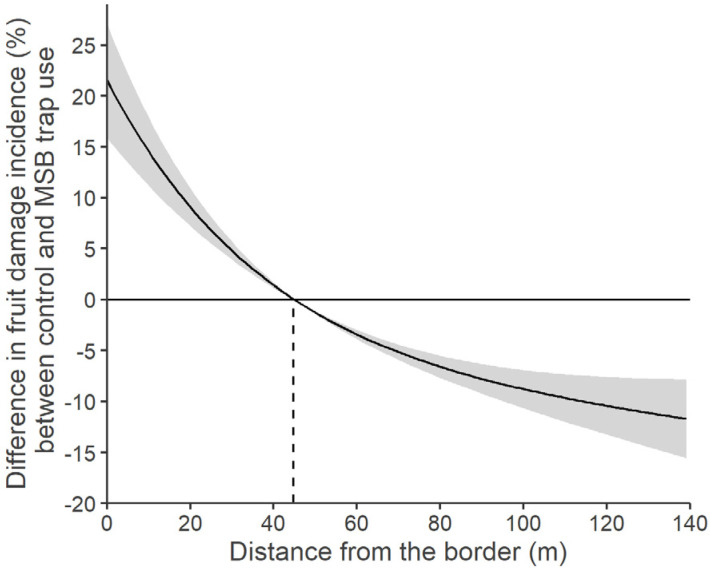
Difference in apple fruit damage incidence in response to the presence of mini–sailboat (MSB) traps baited with *Halyomorpha halys* aggregation pheromone, calculated as the difference between the estimated damage incidence in control plots and in the trap plots, plotted against the distance from the border at the Postal site (South Tyrol, Italy). The point of equal incidence (where no difference exists between control and trap) is 44.82 m (dashed line). Positive values along the curve represent a higher fruit damage incidence in the MSB trap plots, whilst negative values represent a reduction in fruit damage incidence. The grey ribbon reports the estimated standard error.

The point of equal fruit damage incidence between control plots and plots with traps was estimated by the predicted model values. The point at which the predicted incidence was 0 for both control and trap plots was 44.82 m (Figure 7). Before this point, *the fruit damage incidence variation between plots with traps and control plots was negative, signifying higher fruit damage incidence in the presence of traps. After this point, the fruit damage incidence* continued to decrease in the presence of traps, while not in control plots (Figure 7). In the control plots, the total fruit damage incidence was 9.4% ± 8.1. In the plots treated with MSB traps, the total fruit damage incidence recorded within 44.82 m from the border increased to 28.7% ± 27.6, while beyond this distance it decreased to 4.3% ± 5.2.

**Figure 8:**
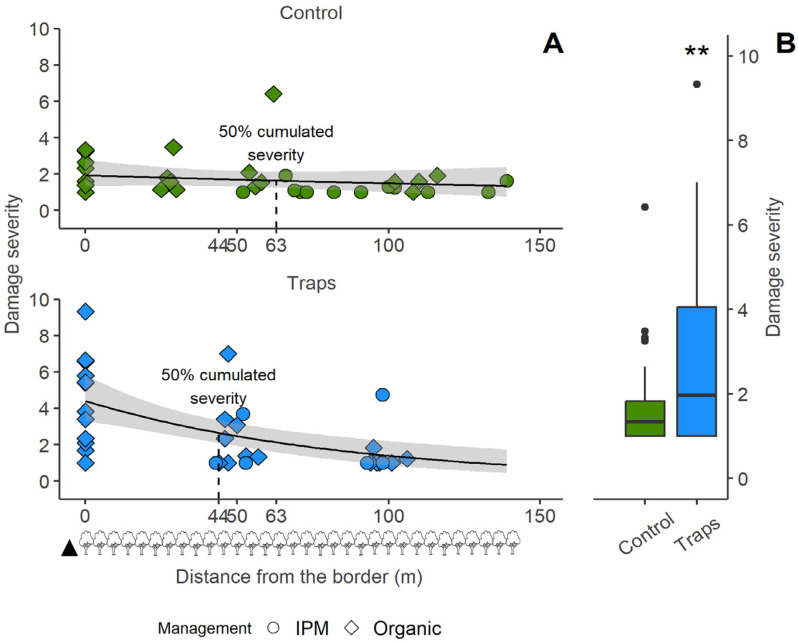
Apple fruit damage severity caused by *Halyomorpha halys* in the control plots and in the plots with mini–sailboat (MSB) traps at the Postal site (South Tyrol, Italy). (**A**) Values plotted against the distance from the orchards border. Dot shape shows whether the parcels were managed organically or according to IPM practice. The lines show the trend, and the grey ribbons display the 0.95 confidence interval around the smooth. The black triangle at the bottom shows the mini–sailboat (MSB) trap location, whilst the tree symbols indicate the plant rows in the apple orchard (space between the rows is ~4 m). Half of the total fruit damage in the plots lies under the curves between 0 and the reported distance of 50% cumulated severity. (**B**) Overall fruit damage severity in control plots and in the plots with MSB traps in the Postal trial (statistical significance: ** *p* < 0.01).

**Figure 9:**
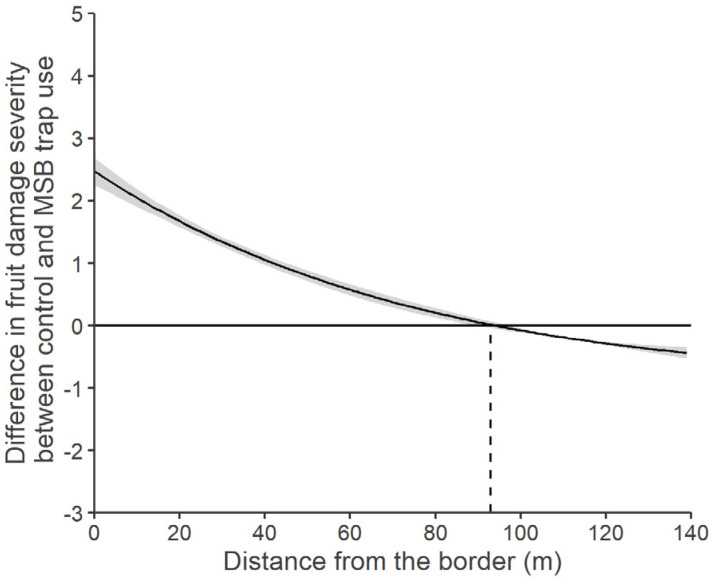
Difference in apple fruit damage severity in response to the presence of mini–sailboat (MSB) traps baited with *Halyomorpha halys* aggregation pheromone, calculated as the difference between the estimated damage severity in control plots and in trap plots, plotted against the distance from the border at the Postal site (South Tyrol, Italy). The point of equal severity (when no difference exists between control and trap plots) is at 93.20 m (dashed line). Positive values along the curve represent a higher fruit damage severity in the MSB trap plots, while negative values represent a reduction in fruit damage severity. The grey ribbon reports the estimated standard error.

The fruit damage severity at the Postal site resembled the fruit damage incidence (Figure 8), without the significance of the terms catch index and apple variety, which were dropped out from the model (model Pearson’s χ^2^ = 55.39, *p* = 0.71). The distance from the border was a more valuable explanatory term for the plots with traps than the distance to the nearest trap, which was then dropped. The fruit damage severity decreased at increasing distances (χ^2^_1,71_ = 6.41, *p* = 0.011). While the fruit damage incidence did not differ between control and trap plots (Figure 6B), the presence of traps increased the fruit damage severity overall (Figure 8B) (χ^2^_1,71_ = 6.58, *p* = 0.009), expressed as the number of injuries per injured fruit with a mean fruit damage severity of 2.92 ± 2.29 in the plots with traps and a mean fruit damage severity of 1.68 ± 1.06 in the control plots. The management approach did not affect the fruit damage severity (χ^2^_1,71_ = 2.16, *p* = 0.141). The distances at which the cumulated fruit damage severity reaches 50% (i.e., half of the estimated total incidence lies between the orchard border and that given distance) slightly varied between control plots and plots with traps, respectively, 63 m and 44 m (Figure 8A). The point of equal fruit damage severity between control plots and plots with traps was estimated by the predicted model values. The point at which the predicted incidence was 0 for both control and trap plots was 93.2 m (Figure 9). Before this point, the fruit damage severity difference between trap plots and control plots was negative, signifying higher fruit damage severity in the presence of MSB traps. After this point, the fruit damage severity decreased in presence of MSB traps (Figure 9).

### 3.3. Analysis of Fruit Damage Caused by H. halys at the Lana Site

**Figure 10:**
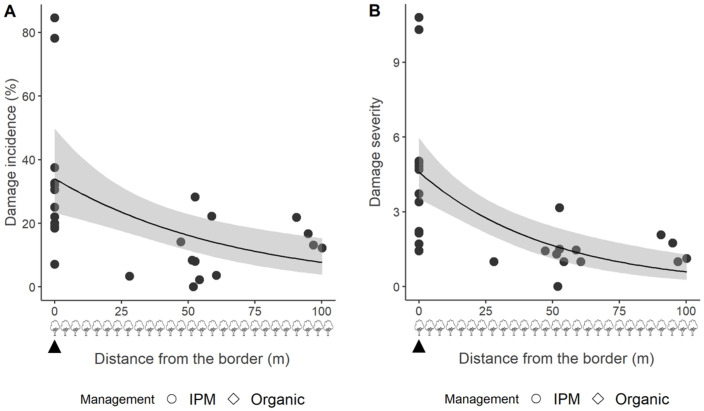
Apple fruit damage incidence (**A**) and severity (**B**) at the Lana site (South Tyrol, Italy) caused by *Halyomorpha halys* plotted against the distance from the orchard border. The lines show the trend, and the grey ribbons display the 0.95 confidence interval around the smooth. The black triangle at the bottom shows the mini–sailboat trap location, whilst the tree symbols indicate the plant rows in the apple orchard (space between the rows is ~4 m).

Regarding the fruit damage incidence, the distance from the border was a better predictor than the distance to the nearest trap, and it was thus a very significant (χ^2^_1,71_ = 38.10, *p* < 0.001) explanatory term (Figure 9), while the catch index and the apple variety, as for the Postal site, were not (model Pearson’s χ^2^ = 35.72, *p* = 0.12). This shows that not the amount of catches, but solely the presence of MSB traps, affects the fruit damage distribution. Similarly to what was observed at the Postal site, the distance from the border (χ^2^_1,71_ = 5.12, *p* = 0.023) was also a better predictor than the distance to the nearest trap in the explanation of the fruit damage severity at Lana (Figure 10) (model Pearson’s χ^2^ = 40.58, *p* = 0.09).

## 4. Discussion

The aggregation pheromone-based lure tested in this study was proven to be very effective in attracting *H. halys* individuals, in agreement with Leskey et al. [18]. Several studies are currently investigating whether and how it can be used for *H. halys* management through behavioral manipulation-based approaches. The most common approach explored so far is ‘attract and kill’ [10,11,12,15]. Our study is the first to examine the use of the *H. halys* aggregation pheromone in combination with MSB traps deployed along the borders of apple orchards, and their impact on the spatial distribution of fruit damage. The new specific design of the MSB trap, featuring a sticky nylon sail and a bin with soapy water to collect insects that fall from above, makes it highly effective for catching flying adult insects. Our results indicate that MSB traps are ineffective for catching *H. halys* nymphs (only 44 nymphs were collected compared to 8322 adults), likely due to the absence of a retention medium at the very bottom of the trap, where nymphs typically arrive after walking. Since nymphs can frequently be observed in the field during the season, and they can cause significant damage to crop like apples, enhancing MSB trap design to capture and retain these nymphal stages more effectively is essential [32]. The MSB traps caught fewer individuals at the beginning of the season (17 May, 8 June, 14 July), reaching the maximum number of catches at the end of the season (26 July, 11 August); this result aligns with the literature describing a low and dispersive *H. halys* population after overwintering and a higher and aggregating population in the second part of the season [2] (Figure 4). The MSB traps were shown to be attractive for both *H. halys* sexes (3695 males and 4627 females). In fact, MSB traps captured adult individuals with a male-to-female sex ratio of 0.85:1, which is consistent with the expected ratio of 0.9:1 observed in surveys of *H. halys* populations [33]. Our results suggest that the differences in the number of males and females captured are not due to variations in trapping efficacy, but rather to the composition of the population. The percentage of *H. halys* catches between the sail and bin showed no significant difference between the two parts of the MSB trap, with ca. 57% of the insects caught on the sticky sail and 43% in the bin. However, while females were recorded in a similar precent on the sail and in the bin, significantly more males were found on the sail (ca. 67%) than in the bin (ca. 32%) (Figure 5). A plausible explanation is that adult insects landing on the sticky sail might later free themselves from the glue. Therefore, still unable to fly, they fall into the bin, where they drown in the soapy water. This phenomenon may have occurred significantly less for males, likely due to their smaller size and lighter weight, making it more difficult for them to dislodge from the glue [34]. This evidence suggests that to maximize the number of insect catches, the MSB trap must be set up with both the sail and bin. In addition, the extremely low number of catches of other pentatomids recorded during our study allows us to consider that the MSB trap, when loaded with the *H. halys* aggregation pheromone, is an extremely selective catch system for this species with negligible side effects for non-targets.

No differences were observed in the number of *H. halys* individuals caught per trap between Postal and Lana, indicating a uniform *H. halys* population density across both sites. Additionally, the similar trap catches within each trial plot suggest a consistent pest presence throughout the apple orchard, with no evident effect of traps on the control plots. Because trap catches were very similar between sites and within the same plot, it was not possible to establish whether higher or lower catches corresponded to higher or lower fruit damage incidence nearby (i.e., ‘catch index’).

Placing MSB traps in small groups with traps spaced 40–50 m apart probably caused them to behave like a single front, emitting aggregation pheromone. The distance from the MSB traps front proved to be a stronger predictor of fruit damage incidence at a given point than the number of insects caught by the nearest trap, highlighting the effect of trap presence on the fruit damage. At the Postal site, fruit damage in the control plots did not show the typical aggregation near the orchard border, as commonly observed with *H. halys* populations [35]. Instead, the damage was spread throughout the orchard, with no clear pattern related to the distance from the borders. This unusual damage pattern can be explained by a strong presence of the pest within the apple orchards, likely due to the second generation developing directly inside, combined with the growers’ adoption of a mild management strategy. In the plots with MSB traps, the fruit damage incidence was strongly influenced by the distance from the border with MSB traps, enhancing the perimeter-driven effect normally observed for this pest species [35]. In particular, we recorded that for the first 44.82 m from the orchard border with MSB traps the fruit damage incidence increased compared with control plots. The point where the fruit damage incidence was equal between control plots and plots with traps was estimated to be at approx. 45 m from the orchard border, after which an actual fruit damage reduction appears. In fact, in the plots with traps, the mean fruit damage incidence was 28.7 ± 27.6 in the first 45 m from the traps and 4.3 ± 5.2 from that point forward. It has to be noted that MSB traps were deployed at approx. 7 m from the apple orchard border; therefore, the fruit damage reduction occurred at approx. 52 m from the effective pheromone source. Our results are in agreement with those presented by Kirkpatrick et al. [36], which state that the aggregation pheromone acts on the *H. halys* adult population within the first 73 m from the traps, in the presence of host plants such as apples. The decrease in damage that we recorded even beyond the 73 m limit may be due to the aggregation pheromone emitted by the large number of insects concentrated in the part of the orchards close to the MSB traps. By integrating the area under the damage incidence distribution curve (Figure 6), it was furthermore possible to calculate the distance from the MSB traps front, within which 50% of the cumulated fruit damage incidence was estimated. This distance falls within the first 25 m from the orchards border in the presence of the MSB traps, while for the control plots it falls at 86 m. Despite this fruit damage shift, it was observed that the mass-trapping method of employing aggregation pheromone in the orchards border did not significantly decrease the total apple fruit damage incidence during pre-harvest, even if a large total number of *H. halys* individuals was caught. In fact, no difference in terms of total fruit damage incidence was recorded in the first site (Postal) between trap plots and control plots. A fruit damage incidence shift towards the traps was also observed in the second experimental site (Lana), confirming what was observed in the other site. Because of the Lana site orchard landscape characteristics, where a control plot was not feasible, we thus lack information on the fruit damage in the absence of traps in this second site. However, the very similar distribution of fruit damage incidence and fruit damage severity recorded in the two sites and the equal number of *H. halys* catches between the sites supports the idea of an analogous fruit damage distribution. In our study, where all the MSB traps were deployed at the border of the apple orchards, the trapping area overlapped with the area of the natural presence of the pest in the apple orchards. It is well known that *H. halys* exhibits perimeter-driven behavior by preferring marginal environments in various crops, including apple crops, and this behavior is often associated with increased fruit damage caused by this insect at the crop border [2]. Our results suggest that the *H. halys* aggregation pheromone enhanced this “border effect” by further limiting the lingering of individuals to a specific area of the orchards. These insects, therefore, have more possibilities to feed on the fruit, increasing the damage. Given these results, there is not enough evidence to determine whether the increase in fruit damage incidence within the first 45 m is only due to an increase in the number of insects attracted by the aggregation pheromone or also to a longer permanence of fewer insects within this area. However, the presence of the MSB traps greatly influenced the fruit damage severity, with a significant increase in plots with traps compared to control plots. Unlike what happened for fruit damage incidence, fruit damage severity increases near the traps but decreases only towards 100 m from the traps, with a distribution of cumulative fruit damage severity similar for plots with traps and control plots. This suggests, in agreement with previous works [10,35], in which *H. halys* foraging behavior was influenced by the presence of the aggregation pheromone, an increase in the overall acceptability of the host plant resource and then a reduction in *H. halys* dispersal from them.

Our results suggest that the use of aggregation pheromone for *H. halys* management, despite not being effective for MT, shows promise as a tool for enhancing IPM-CPR techniques. Aggregating the *H. halys* population to a narrower area alongside the orchard border, thus reducing their dispersion, makes it possible to target insecticide applications to only a limited part of the apple block, maximizing the sprays’ efficacy. In addition, concentrating damaged fruit in a few border rows, rather than having it evenly across the orchard, can reduce sorting time and speed up harvesting in unaffected areas. This shift in damage to the borders simplifies the harvest process by decreasing the time needed to separate damaged from undamaged fruit, even in heavily infested orchards. However, this potential benefit is strongly linked to the shape and size of the apple orchards. Our study considered only extensive apple orchards, resulting in a border to interior ratio that favored the interior, which is the setting where border treatments are most effective.

Further studies should focus on comparing the effectiveness of localized insecticide applications targeting the aggregated *H. halys* population in the first rows of the apple orchard near MSB traps, with the classical IPM-CPR technique, the traditional “attract and kill” approach described by Morrison et al. [10], or the use of LLINs [12,37]. Furthermore, the effectiveness of this approach should be evaluated, repeating such trials over multiple seasons. The traps could offer valuable insights into pest population size before and after insecticide treatments. The cost–benefit evaluation of the MT approach using MSB traps must be carefully considered, since the costs related to this technique must return to the growers as a reduced fruit damage level or as a reduced use of insecticides in order to implement a sustainable IPM strategy for *H. halys*. Finally, mass-trapping approaches can only be implemented when sufficiently powerful attractants are available. These attractants must be capable of competing with the food stimulus within the agroecological landscape. This could be achieved by seeking chemical signals completely different from the aggregation pheromone (e.g., volatile organic compounds emitted by very attractive host plants), which should reduce the time the *H. halys* individuals remain within the apple orchards, and thus the feeding opportunities. Therefore, another promising follow-up of this study is to explore the combined use of aggregation pheromone and attractive host plant volatiles acting as kairomones, to potentially enhance the mass-trapping activity of the MSB traps.

In summary, our study demonstrates the effectiveness of MSB traps combined with the *H. halys* aggregation pheromone in attracting a large number of *H. halys* adults. However, the traps were less effective in capturing *H. halys* nymphs, indicating the need for design improvements to better retain nymphal stages. Our results also revealed that despite the fruit damage shift caused by the MSB traps toward the orchard borders, the fruit damage incidence overall was not significantly reduced compared to the control areas. Additionally, the severity of damage increased near the MSB traps. The spatial distribution of damage suggests that the traps concentrated *H. halys* populations within a narrow zone along the orchard perimeter, intensifying the “border effect” typically observed with this pest. For this reason, this trapping method shows promise for enhancing strategies such as IPM-CPR. These findings, in fact, highlight the potential of MSB traps for targeted insecticide applications along orchard borders, reducing the need for widespread treatments. Further research should focus on testing the efficacy of localized pest control measures in different settings and exploring the use of additional attractants, such as volatile organic compounds, to improve mass-trapping performance.

## Data Availability

The original contributions presented in the study are included in the supplementary material, further inquiries can be directed to the corresponding author.

## Supplementary Materials

The following supporting information can be downloaded at https://www.mdpi.com/article/10.3390/insects15100791/s1, Table S1: The 107 fruit damage assessment points of the trial conduced in 2023 in the apple orchards at the sites of Postal and Lana, South Tyrol, Italy. Fruit damage incidence and fruit damage severity; Table S2: Total *Halyomorpha halys* catches for the trial conduced in 2023 in the apple orchards at the sites of Postal and Lana, South Tyrol, Italy. With the number of adults female and male, and nymphs. Data are showed per date and per trap. Table S3: Summary of the final reduced model fit of fruit damage incidence caused by *H. halys* at the Postal site (South Tyrol, Italy).
