# Supplementary material for: Impact of Aggregation Pheromone Traps on Spatial Distribution of Halyomorpha halys Damage in Apple Orchards
Source: Insects. 2024 Oct 11;15(10):791. doi: 10.3390/insects15100791 (PMC11509157; doi:10.3390/insects15100791)
Supplement: Supplementary file 1 [file insects-15-00791-s001.zip › S3_Carnio_etal_rev.pdf]

Table S3: Summary of the final reduced model fit of fruit damage incidence caused by *Halyomorpha halys* in Postal site (South Tyrol, Italy).

**Variable**	**Estimate**	**SE**	**z**	***p***
Intercept	1.070	0.430	2.480	0.013
Traps	0.370	0.630	0.590	0.550
Management organic	0.991	0.378	2.620	0.008
Distance from border	0.008	0.003	1.250	0.083
Distance to the nearest trap	-0.003	0.003	-1.220	0.220
Traps * Management organic	0.989	0.568	1.720	0.081
Traps * Distance from the border	-0.028	0.005	-5.190	<0.001
Traps * Distance to nearest trap	0.036	0.016	2.210	0.026
